# Evaluating RGB Imaging and Multispectral Active and Hyperspectral Passive Sensing for Assessing Early Plant Vigor in Winter Wheat

**DOI:** 10.3390/s18092931

**Published:** 2018-09-03

**Authors:** Lukas Prey, Malte von Bloh, Urs Schmidhalter

**Affiliations:** Chair of Plant Nutrition, Technical University of Munich, 85354 Freising, Germany; prey@wzw.tum.de (L.P.); malte.von.bloh@tum.de (M.v.B.)

**Keywords:** early vigor, image segmentation, high-throughput, hyperspectral vegetation indices, contour map analysis, Greenseeker, nitrogen status, precision farming, phenomics

## Abstract

Plant vigor is an important trait of field crops at early growth stages, influencing weed suppression, nutrient and water use efficiency and plant growth. High-throughput techniques for its evaluation are required and are promising for nutrient management in early growth stages and for detecting promising breeding material in plant phenotyping. However, spectral sensing for assessing early plant vigor in crops is limited by the strong soil background reflection. Digital imaging may provide a low-cost, easy-to-use alternative. Therefore, image segmentation for retrieving canopy cover was applied in a trial with three cultivars of winter wheat (*Triticum aestivum* L.) grown under two nitrogen regimes and in three sowing densities during four early plant growth stages (Zadok’s stages 14–32) in 2017. Imaging-based canopy cover was tested in correlation analysis for estimating dry weight, nitrogen uptake and nitrogen content. An active Greenseeker sensor and various established and newly developed vegetation indices and spectral unmixing from a passive hyperspectral spectrometer were used as alternative approaches and additionally tested for retrieving canopy cover. Before tillering (until Zadok’s stage 20), correlation coefficients for dry weight and nitrogen uptake with canopy cover strongly exceeded all other methods and remained on higher levels (R² > 0.60***) than from the Greenseeker measurements until tillering. From early tillering on, red edge based indices such as the NDRE and a newly extracted normalized difference index (736 nm; ~794 nm) were identified as best spectral methods for both traits whereas the Greenseeker and spectral unmixing correlated best with canopy cover. RGB-segmentation could be used as simple low-cost approach for very early growth stages until early tillering whereas the application of multispectral sensors should consider red edge bands for subsequent stages.

## 1. Introduction

Increasing the efficiency in using fertilizer and pesticides in cropping systems has become a crucial challenge in current crop production. In recent years, spectral sensing has become a versatile tool for evaluating crop stands and determining fertilizer demand [1,2]. Sensors can be used either offline to support nitrogen management, or online, enabling fully automatic site-specific application of fertilizers [3,4]. Multispectral remote sensing is widely used for crop monitoring [5]. However, transferring the physical signal of the visible and infrared spectra into ready-to-use recommendations on fertilizing rates is not trivial. Fertilizer algorithms depend on the characteristics of crops and cultivars with respect to the morphology such as leaf angle, the target yield and most-importantly, on the current growth stage [6,7,8].

Canopy sensing is not recommended in early growth stages, due to distortion by soil background scattering in sparse canopies [9,10], which results in mixed soil-plant pixels. Therefore, only rare attempts were done to unlock the potential of a sensor-guided fertilization at the early stages before tillering. To overcome such difficulties, partly oblique sensor views have been recommended for spectral reflectance sensing [9,11], thus however excluding the direct comparison to mostly Nadir-based imaging.

A large number of vegetation indices has been developed over time to predict vegetation traits based on different spectral parameters, but their usage is sensor-dependent and has rarely been evaluated at early growth stages [12,13]. Frequently used spectral vegetation indices use wavebands of the red and near-infrared (NIR) spectrum to predict key traits such as biomass or nitrogen (N) content. The widespread normalized difference vegetation index (NDVI) is based on the visible red and NIR spectrum. The red-edge inflection point (REIP) and the normalized difference red edge index (NDRE) both use wavebands that are closer to the red edge [14,15,16]. The water band index (WBI), that uses only of two bands in the NIR range, was found to be less prone to saturation in dense canopies [17,18]. The photochemical reflectance index (PRI) only uses wavebands in the visible green spectrum, however. It was developed to detect the photosynthetic capacity, but is also sensitive to pigment content [19,20]. An index, that has been developed specifically for the measurement of early plant development is the early plant vigor index (EPVI) [21], which uses three single wavebands from the red, red edge and NIR spectrum. Red edge-based indices including the modified chlorophyll absorption in reflectance index (MCARI) and the MERIS terrestrial chlorophyll index (MTCI) were found to be sensitive to plant N content also in denser canopies [22,23].

Brightness and surface structure of the soil impact spectral measurements in sparse canopies [10,24,25], influencing early estimation of biomass in wheat [26]. Soil-adjusted vegetation indices (SAVI) were developed for reducing sensitivity to soil background radiation [10,27] and were found to be advantageous over NDVI for leaf area index estimation in early stages [28]. For a general overview of factors influencing spectral sensing see [12].

Alternatively, the total spectral information can be included through spectral unmixing, which represents the spectral signature as mixed information of two or more surface types, which are referred to as endmembers [29]. Examples of using spectral unmixing for plant modelling can be found in [30,31]. Fluorescence measurement was recommended as alternative to reflectance-based sensing due to being less influenced by soil background and plant architecture [9,32].

Early plant development is an important trait influencing early N-uptake, potential weed suppression and water use efficiency, all of which can be related to final yield potential. Detecting such traits is equally relevant in precision farming for potentially guiding early fertilization as well as for phenotyping for identifying superior genotypes [33,34,35,36,37,38].

In remote sensing, image-based techniques involving only few wavebands have been prevalent for a long time [39]. However, until now, in applied proximal sensing, image-based techniques for characterizing plant canopies are less common. Nevertheless, a substantial number of studies has shown that digital imaging can be used to retrieve different plant parameters. Digital images are composited by pixels which are a combination of the color channels red-green-blue (RGB). Similar to spectral data, indices can be calculated from these channels [40]. Pagola et al. used digital image analysis to develop a greenness index to estimate yield through the estimation of the N-content of barley leaves and reported a negative correlation between both traits [41]. Other authors [42] reported similar or better correlations with N status compared to spectral approaches when color analysis was reduced to segmented RGB images. Often, the red and green channels were found to be most informative as compared to the blue channel [42].

Various attempts have been undertaken to use simple RGB-based pixel analysis to retrieve fractional canopy cover showing acceptable correlations up to r = 0.87 with aboveground biomass [43,44]. Comparing image segmentation to Greenseeker-NDVI for phenotyping early vigor in wheat, image-based canopy cover gave better correlations with leaf area index across a leaf area index range from 0.5 to 2.5 and was well associated with PAR absorption and dry matter [38]. In early growth stages, combining multispectral imaging with NDVI was recommended [45]. Image-based canopy cover was found to correspond to plant traits like biomass and leaf area index [46,47]. These authors concluded that digital image analysis can deliver high quality results until canopy closure at flowering whereas it is not able to resolve the three-dimensional canopy structure [47].

While biomass, nutrition or water status are key target traits in plant nutrition, digital images could also be used to quantify the amount or type of weed, to detect plant diseases and to estimate soil evaporation [38,48,49,50,51]. See [5,52] for further reviews on current imaging applications in plant phenotyping and monitoring.

Image-based techniques could hold multiple advantages such as lower sensor costs and robustness when consumer-grade cameras can be used [42]. Furthermore, methods tested on ground-based cameras can be transferred to UAV-based applications for increasing the throughput [53,54]. However, the ground resolution will remain an important issue to be addressed.

The objective of this study was to evaluate the performance of a consumer-grade camera for detecting biomass, N-uptake and N-content at four early plant growth stages (Zadok’s stages 12–32), corresponding to leaf development until early stem elongation, in comparison to a simple active Greenseeker sensor and vegetation indices of a passive hyperspectral spectrometer in winter wheat. It is hypothesized that digital images will show better results in the early stages, while spectral remote sensing will be negatively influenced by soil backscattering. With increasing canopy cover (CC) and plant growth, spectral sensing could improve and replace digital-image analysis due to saturation of the green-pixels in the imaging approach.

## 2. Materials and Methods

### 2.1. Experiment and Measurements

The experiment was established as a split-split plot design with three replicates at the Dürnast research station of the Technical University of Munich located in South-East Germany (48.406 N, 11.692 E) and consisted of two parts: The first block was sub-divided into two main blocks with different N fertilizer application rates with N1 receiving 30 kg N ha^−1^ and N2 receiving 60 kg N ha^−1^ for the first nitrogen dressing. The line winter wheat cultivars Anapolis, and Mulan and the hybrid wheat cultivar Hybery were grown on the sub-plot. Anapolis was distinguished by pronounced planophile growth until tillering. The second block was divided into three sowing rates including 150, 250 and 350 kernels m^−^² and the cultivars Anapolis and Hybery on the sub-plot. The field was comprised mostly of a homogeneous Cambisol with a silty clay loam soil textural class. The average annual precipitation in this region is approximately 800 mm with an average annual temperature of 8 °C. The previous crop was grass-clover.

Plant sampling and sensor measurements were conducted at four dates during the early growing season in 2017 at the beginning of vegetation from March 15 to May 3, corresponding to Zadok’s growth stages 12–32 (Table 1). Plant sampling was conducted in three replicates for each treatment combination, resulting into 36 data points per sampling date. At each sampling date, a sampling area of 0.5 m × 0.5 m was marked. RGB images were taken with a consumer-grade D5100 camera (Nikon, Chiyoda, Japan) equipped with an AF-S DX NIKKOR 18–105 mm objective (Nikon, Chiyoda, Japan) at the height of approximately 1.6 m under diffuse ambient light conditions. Only on date 3, an umbrella was hold above the camera for ensuring homogeneous lightning conditions due to clear sky conditions. The camera settings were adjusted manually on each measurement day but kept constant during measurements due to stable illumination conditions. In order to reduce angular distortion effects, focal length was kept minimum at 18 mm, resulting in an image angle of 76°. Exposure time was adjusted to achieve slight overexposure, which improved image segmentation. Images were saved in the jpeg format.

Afterwards, spectral measurements were taken using the GreenSeeker^®^ Handheld Crop Sensor (Trimble, Sunnyvale, CA, USA) for retrieving NDVI, and from the bidirectional hyperspectral passive spectrometer Handyspec (tec5, Oberursel, Germany). The Handyspec yielded spectral readings from 300 nm to 1140 nm at a nominal resolution of 3.3 nm. It is equipped with two silicon diode array spectrometers (Zeiss, Oberkochen, Germany) which measure simultaneously downwelling and upwelling radiation. It was calibrated prior to the measurements using a polytetrafluoroethylene (PTFE) white standard (Spectralon Target, Labsphere, Inc., North Sutton, NH, USA).

The actively measuring Greenseeker sensor emits light by two diodes and detects the reflection in the visible range around 656 nm and in the NIR range at 774 nm, with a band width of ~25 nm in both bands. The readings are directly provided as NDVI value. Both sensors were activated for approximately 5 s and delivered averaged sensor readings.

Both sensors were held in nadir direction over the center of the sampling area. Hold at 60–70 cm above the canopy, the oval field of view of the Greenseeker stretched in parallel to the sowing direction has a length of 25–30 cm. For the Handyspec, the aperture angle of 25° resulted in a circular field of view giving a diameter of 25–30 cm at the same measurement height.

Thereafter, total aboveground biomass of the marked sampling area was manually sampled and washed for removing adherent soil particles. Plant samples were dried for two days at 50 °C and weighed for the determination of the dry weight (DW) and were finely ground. Biomass N content (NC) was determined by NIR-spectroscopy (Rapid Content Analyzer, Foss, Hilleroed, Denmark). N-uptake (Nup) was calculated by multiplying DW with NC.

### 2.2. Data Analysis

Statistical analysis was conducted in the open source software R 3.4.2 [55]. A two-way ANOVA was performed on destructively sampled traits within both main plots of the trial due to unbalanced data of the overall experiment.

RGB images were subjected to color-based image segmentation using the open source software ImageJ [56], similar to the procedure as described by [38]. Images were manually cropped to the predefined rectangular sampling area, which had been tagged in the field with colored wooden sticks in the four corners. Hence, the images were of quadratic shape delivering approximately 2400 by 2400 pixels. Vegetation pixels were separated from background soil in the HSV-color space mainly based on differentiation in the HUE and Saturation channels. Settings were manually adapted for the set of pictures of each sampling date on a few images and kept constant for the remainder of the image set. Thereafter, the relative amount of green pixels among all pixels, referred to as canopy cover (CC) was extracted.

From the Greenseeker sensor, only one default NDVI-index was available. The hyperspectral Handyspec data was exploited in different ways. Several preselected narrow-band vegetation indices were calculated using the passive spectral data (Table 2): The standard NDVI index was complemented by a red-edge NDVI using a NIR band closer to the red edge (NDRE) as well as the MTCI and REIP, which were deemed suitable for N detection due to bands close to the red-edge. The MCARI was included as an index designed for leaf chlorophyll detection with potential for estimating the leaf N content. The PRI was evaluated due to using only bands in the visible spectrum in comparison to the camera RGB-bands. In contrast, the water band index WBI was included due to using only two NIR bands. Furthermore, ATSAVI and EPVI were included. Simple linear spectral unmixing was applied on the hyperspectral data using two endmembers corresponding to bare soil and dense vegetation, which were manually selected for each measurement day [29]. The method regresses the observed mixed canopy spectrum on its two endmembers. The spectra of the endmembers were used as predictors in a multiple regression in *R*. The estimated canopy coverage was calculated as ratio of the regression coefficient for dense vegetation to the sum of the regression coefficients of both endmembers.

The image-based CC, the Greenseeker NDVI, all pre-defined, established spectral indices and the spectral unmixing estimate for canopy coverage obtained from the Handyspec measurements were used for testing simple linear correlations with the target traits DW, Nup and NC for each sampling date. Likewise, considering CC as target trait, the Greenseeker and Handyspec readings were tested for correlations with CC. Since the direction of the relationship between sensor methods and the target traits is often not of interest, the squared correlation coefficient (R²) was reported for more direct comparison of different methods.

Mean and coefficient of variation (CV) of the R²-values from the tested sensor methods were retrieved over the four measurement dates for evaluating the level and stability of the models.

In addition to established spectral vegetation indices, pairwise normalized difference indices were calculated from all possible band combinations and also tested in correlation analysis for each date*trait combination using the hsdar-package in R [61]. The spectral range at 300–400 nm was removed due to scattering. This resulted in an array containing four contour map matrices with squared Pearson correlation coefficients (R²) per trait. As for the other methods, maximum R² values were extracted for each matrix together with the included wavelengths. Mean values and coefficient of variations (CV) were calculated along the temporal dimension.

## 3. Results

### 3.1. Seasonal Development of Reference Traits

In the sowing density block, no significant cultivar effects were observed for the four target traits for any of the measurement dates (D) (not shown). Differences between sowing densities were observed on all days for DW and CC, for Nup on all days except on D4 but only on D4 for NC. In the nitrogen fertilization block, no fertilization effect was found. For cultivar effects, only Mulan showed significantly lower CC than Hybery and Anapolis on D3 and D4.

**Figure 1:**
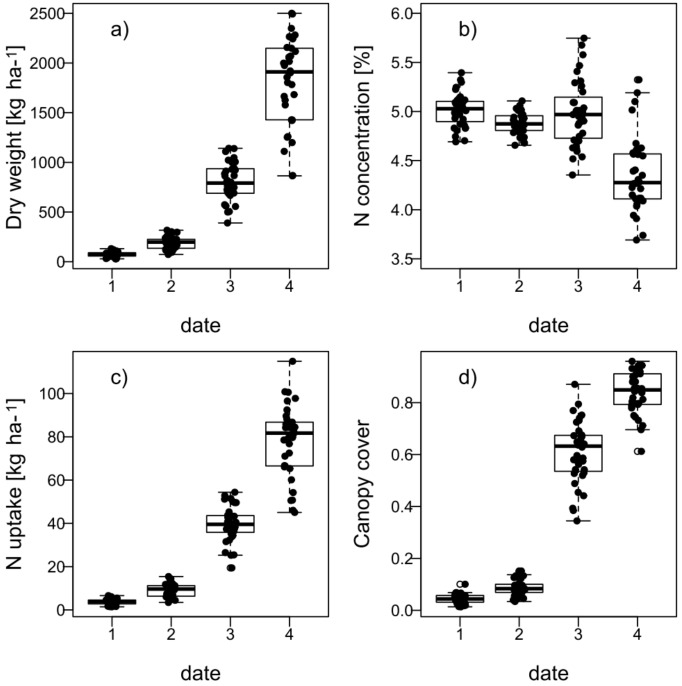
Development of reference data over four measurement dates: Dry weight DW (**a**), nitrogen content NC (**b**), nitrogen uptake Nup (**c**) and canopy cover CC (**d**) (*n* = 36).

Still, substantial differences between experimental plots were found. All traits increased exponentially over the measurement dates with the exception of NC (Figure 1). DW and Nup followed a similar pattern. Average NC remained almost stable until D3 at approximately 5% before dropping to 4.6% but differentiated more strongly from D3 onwards.

**Figure 2:**
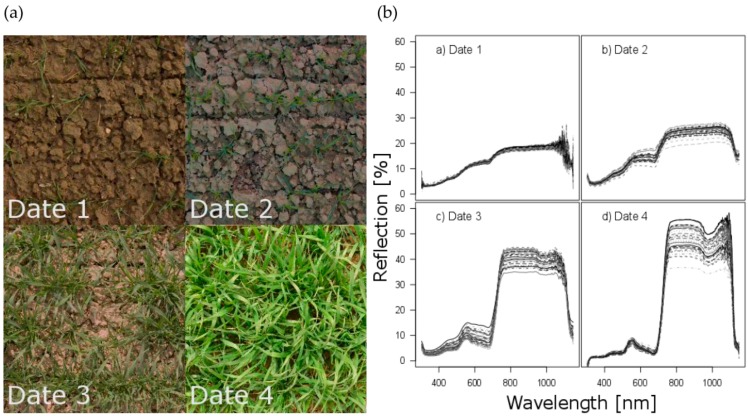
(**a**) Example of canopy development of a selected plot with cv. Hybery at N-level 2. (**b**) Development of spectral reflection of all experimental plots for four measurement dates.

CC however approached a sigmoidal shape from the beginning of vegetation (D1, seedling growth), when on average only 4.5% of the ground was covered by the canopy, until early stem elongation (D4, average of 84%, Figure 1d and Figure 2a). DW and Nup increased from on average only 73 to 1812 kg ha^−1^ and 3.7 to 78 kg ha^−1^, respectively. DW and Nup were always closely correlated (R² = 0.91–0.99) whereas NC showed medium negative correlations with DW on D3 and D4 and with Nup on D4, indicating a strongly dominating effect of DW on Nup (Table 3). The sparse canopy on D1 (Figure 2a) was evidenced by the flat reflection curve (Figure 2b). The reflection in the near infrared (NIR) range successively increased and differentiated more with ongoing plant development. The first day was affected by stronger scattering above 1000 nm. By D4, most plots had almost reached canopy closure, evidenced by the pronounced reflectance spectra.

### 3.2. Correlation Results for Evaluated Sensors and Target Traits

**Figure 3:**
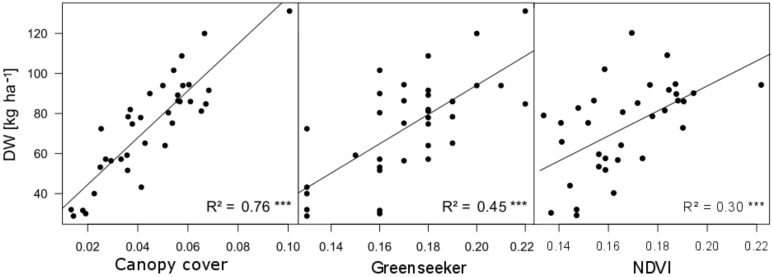
Relationship of selected sensor methods with DW for first sampling date (03/15/2017).

Linear correlations were tested between image-based canopy cover (CC), the Greenseeker-NDVI as well as selected vegetation indices and spectral unmixing from the hyperspectral data, with the reference traits DW, NC and Nup for all dates (Table 3). In addition, CC was included as reference trait and correlated with the sensor values of the Greenseeker and Handyspec. The strongest differentiation between sensor methods was observed on the first date (D1). All vegetation indices calculated from the hyperspectral sensor performed poorly with R² values peaking at 0.31 for DW (NDRE), 0.34 for Nup and 0.25 for CC (NDRE, NDVI, ATSAVI for both traits). WBI, PRI and spectral unmixing did not have significant relationships (not shown). For all hyperspectral vegetation indices, relationships with CC were even weaker than with DW and Nup. Surprisingly, Greenseeker outperformed all hyperspectral vegetation indices for all traits (R² = 0.45 with DW and Nup and R² = 0.49 with CC). However, the best correlation coefficients were found using canopy cover in relationship to DW and Nup (R² = 0.76 and 0.78, respectively) (Figure 3).

No significant correlations with N content (NC) were found using any of the tested established methods, both for D1 and D2. Correlations strongly increased for most other sensor method/rait combinations on D2. Again, NDRE yielded best correlations with DW and Nup (R² = 0.75 and 0.76, respectively) but was slightly outperformed by the spectral unmixing method for CC (R² = 0.56). Similar to D1, differences in R² values of NDRE and ATSAVI were small but EPVI performed less well (R² = 0.69 for Nup). However, models from WBI, PRI and REIP provided again lower correlations compared to the best indices. This was now also the case for the Greenseeker sensor (R² = 0.54 for Nup) in spite of still increasing R² for DW and Nup but decreasing R²-values for CC in comparison to D1. In contrast to all hyperspectral indices, CC used as sensor method followed an opposite trend and reached lower correlations than on D1, both with DW and Nup (0.65 and 0.67).

Contrasting to D1 to D2, the overall index ranking changed and was not similar for all target values on D3, even if the level of correlations remained comparable. REIP, which strongly improved from D2, yielded now best R²-values with DW (0.77) and was slightly outperformed for NC by MTCI (0.35). However, these indices ranked not among the best indices for CC. The indices Greenseeker and PRI reached best correlations for CC (R² = 0.73), followed by ATSAVI and EPVI.

On D4, correlations decreased for almost all sensor methods for DW and Nup compared to D3, with PRI, EPVI, ATSAVI, NDVI, MCARI and spectral unmixing showing the strongest reduction. While on D3, NDVI, NDRE, WBI, and CC formed a rather homogenous group with similar R² values for DW and Nup, NDVI yielded now weaker correlations. Still, REIP reached best correlations both with DW and Nup (0.67 and 0.58, respectively). However, for estimating CC, spectral unmixing and Greenseeker (both 0.70) reached the best correlations on this day. While the ranking of sensor methods was close between Nup and DW for all days (R² between correlations for both traits > 0.93), the index ranking substantially differed between CC and DW for D3 and D4.

### 3.3. Contour Map Analysis

**Figure 4:**
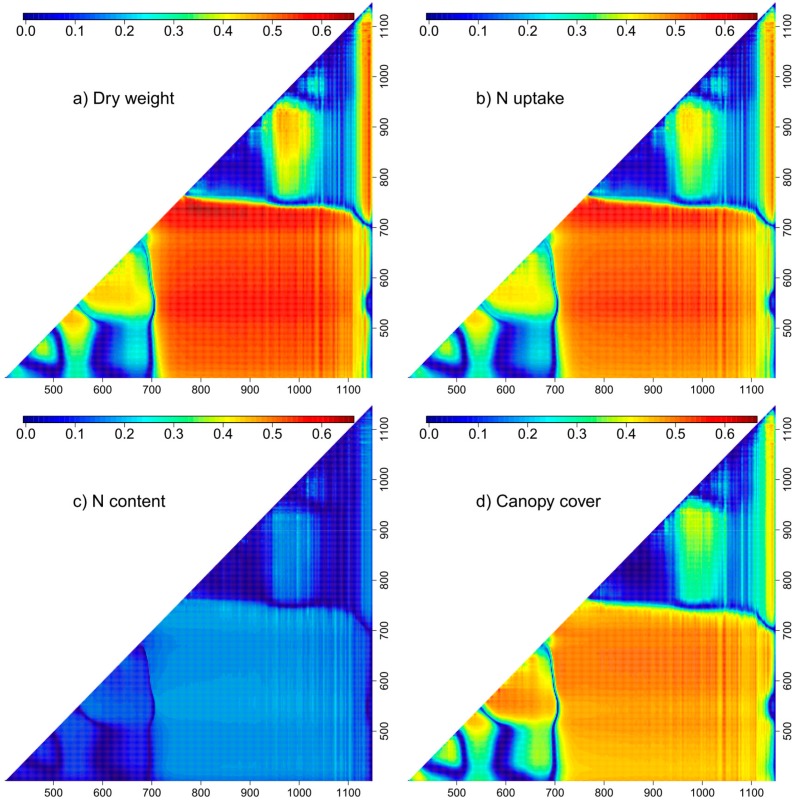
Contour map matrices: Mean squared Pearson correlation coefficients (R²) calculated across the contour maps of the four measurement dates for four target traits. Axis denote spectral bands (nm).

The best hyperspectral normalized difference indices found in the contour maps revealed a higher potential for the estimation of all traits compared to established hyperspectral vegetation indices. However, the relative increase in R² was stronger for measurement days and traits with lower correlation level from established sensor methods (D1 and NC, respectively). Similar to the pre-defined vegetation indices, contour maps averaged over four measurement days for DW and Nup showed similar correlation patterns (Figure 4, Table 3). Moreover, the optimum indices extracted from the correlation matrices averaged across the four measurement days were almost identical for both traits (792 nm; 736 nm for DW and 794 nm; 736 nm for Nup).

For DW and Nup, a broad field of combinations of VIS/NIR bands yielded on average enhanced correlations (R² > 0.5), which however were exceeded by red edge/NIR-combinations (Figure 4). The pattern for NC was comparable but on a distinctively lower level, peaking at an R² value of only 0.22 (996 nm; 924 nm).

For estimating CC, the pattern was similar as for DW, but without peaks in the red edge/NIR range. In contrast to the other traits, a second peak was visible for combinations in the red/red and red/green range (500–680 nm), with the maximum R² (0.55) reached by a narrow-banded spectral combination at 584 nm; 582 nm.

**Figure 5:**
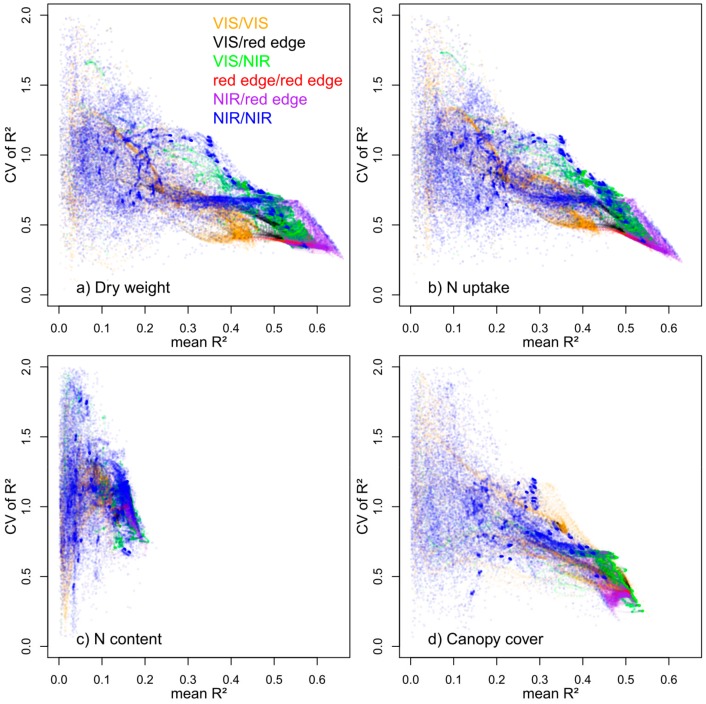
Coefficient of variation (CV) of R² plotted against mean R² over four measurement dates, colored by wavelength groups. The visible (VIS) range was defined as 400–680 nm, the red edge as 680–740 nm and the near infrared (NIR) range as 740–1140 nm.

The stability of index correlations from the contour maps analysis over the four measurement dates differed between spectral regions and was generally inverse to the mean R² (Figure 5). For DW and Nup, on average best contour map-indices from NIR/red edge combinations mostly also showed lowest coefficients of variation (CV) over time, thus data clouds tapered arrow-like into a narrow zone of a few number of vegetation indices with maximum R² but with low CV. However, vegetation indices with low average R² differed strongly in CV. Many NIR/NIR combinations were particularly affected by scattering effects (Figure 4), that went along both with higher variation between measurement days and between indices. Thus, these indices delivered a large spread in mean R² and higher CV for given mean R² values compared to VIS/VIS, VIS/red edge and especially the mostly stable red edge /red edge indices. A similar effect was visible also for VIS/NIR bands.

The best vegetation indices for DW and Nup only comprised NIR/red edge combinations (purple) and a few NIR/NIR combinations (blue) followed by the VIS/NIR group (green) and combinations of two red edge bands (red), whereas VIS/red edge (black) and VIS/VIS (orange)combinations dropped in mean R². Compared to NIR/NIR and VIS/VIS indices, all combinations including red edge bands differed substantially less among each other in mean R².

Unlike for DW and Nup, for CC, overall best correlations paired with lowest CV were found from VIS/NIR and VIS/VIS combinations. However, in contrast to DW and Nup, a clear narrowing at the bottom of the figure is missing.

**Figure 6:**
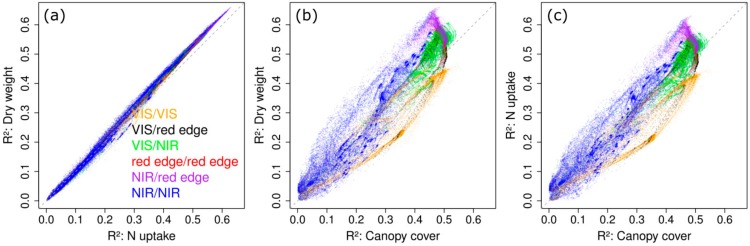
DW~Nup (**a**) DW~CC (**b**) and Nup~CC (**c**). Mean R² values across dates for DW, Nup and CC plotted against each other, colored by wavelength groups.

The mean R² values as depicted in Figure 4 were plotted against each other for enabling pairwise comparison of the relative performance of index groups between traits (Figure 6). As observed for pre-defined vegetation indices (Table 3), correlations were almost identical for DW and Nup, even if the top third of the indices performed slightly better for DW. Correlations for CC differed more strongly from those of DW: NIR/red edge-VI (purple) yielded better results for DW than for CC by up to 0.12 whereas most VIS/VIS combinations performed better for CC than for DW. For DW compared to NC, all index groups performed clearly better.

### 3.4. Correlation Level and Model Stability Over Time

**Figure 7:**
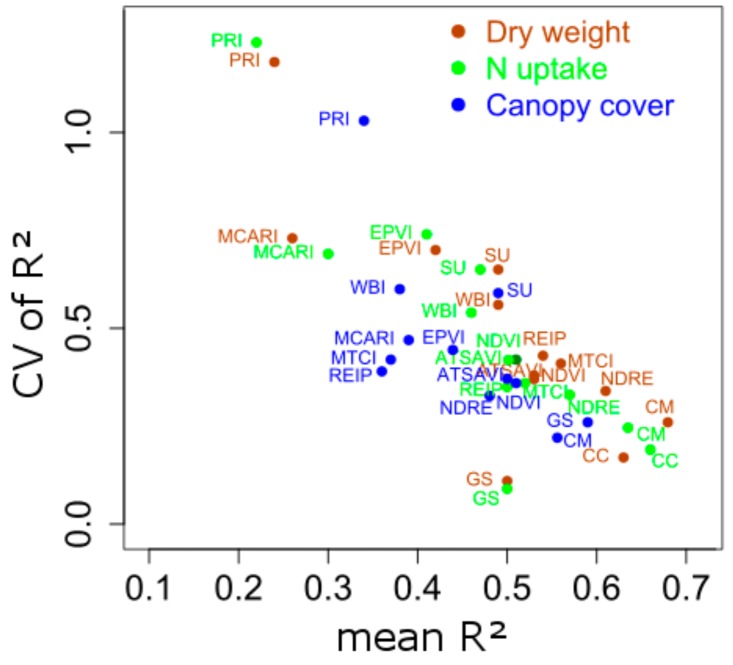
Coefficient of variation (CV) of R² plotted against mean of R², calculated across four measurement days. Data of non-significant correlations was included for the calculation.

Mean R² values of pre-defined and best contour map indices as listed in Table 3 are depicted in relationship to their CV (Figure 7).

As for all contour map indices (Figure 5), a negative relationship between mean and CV of R² could be observed. PRI and MCARI, both indices that include only VIS-bands, represent outliers with mostly unstable and weak performance. The best contour map index yielded the best mean correlation for DW only. For DW (brown) and Nup (green), Greenseeker reached lowest CV, yet with a markedly lower mean R². Canopy cover used as a sensor method, however, reached similar mean R² values as respective contour map indices for DW and Nup with still lower CV values. Amongst the hyperspectral vegetation indices, NDRE performed best for DW and Nup, followed by the close group of NDVI, ATSAVI, REIP and MTCI. WBI and spectral unmixing reached lower mean R² values with higher variation from spectral unmixing, followed by EPVI. For estimating CC (blue), Greenseeker gave the best mean and CV of R² followed by the best contour map index and the similarly performing NDVI, ATSAVI, and NDRE as well as spectral unmixing, which however was clearly less stable over time.

## 4. Discussion

The main objective of this study was to evaluate the performance of a consumer-grade camera for detecting biomass and N-uptake at four early growth stages (Zadok’s stages 12–32) in comparison to an active Greenseeker sensor and different vegetation indices of a passive hyperspectral spectrometer in winter wheat.

### 4.1. Seasonal Plant Development

Apart from the sowing density trial, mostly no significant treatment effects were found during early plant development. The sampling period was characterized by dry weather with little precipitation within three weeks following fertilization and cold temperature when higher amounts of rain fell from March 26 onwards. Thus, plant growth and the effect of N fertilization was limited whereas differentiation on plot level was also driven by heterogeneous plant development in the trials.

### 4.2. Differentiation of Correlations by Traits and Sensor Methods

From all sensor methods, largely similar correlations were found for DW compared to Nup, what can be attributed to the close correlation between both target traits on all days, whereas little variation in Nup was due to differences in NC. However, as reported for later stages as dilution effects [62,63], an increasing trade-off between DW and NC was observed on D3 and D4. This indicates that Nup during early development was increasingly limited by the N availability but rather associated with development of biomass on D1 and D2. Since NC was detected by different sensor methods only on days when (negative) correlations between NC and DW were found, we assume that the correlations found for NC originate indirectly from the detection of DW only. Consequently, the pigment-related MCARI, that should be most sensitive to NC amongst tested vegetation indices, failed.

The correlations of image-based canopy cover (CC) with DW and Nup decreased over time. With the plots approaching canopy coverage on D4, CC became saturated whereas DW and Nup increased exponentially in this phase. Therefore, CC was found less suitable for the estimation of traits as soon as canopy development shifted into the vertical direction [46,47].

On the other hand, CC clearly outperformed all other sensor methods on D1 and could still compete with most vegetation indices on D2, thus substantiating the initial hypothesis that spectral sensing is limited through soil background scattering [16,27,64]. The ATSAVI, however, optimized for overcoming this effect, provided only similar correlations with all traits compared to the NDVI on D1 and D2, which may be due to the standard adjustment factors that were used and may not be adapted to the specific soil conditions. Even if contour map indices indicated possible improvements, they could not even nearly compete with the imaging method on D1.

Surprisingly, the Greenseeker-NDVI exceeded all hyperspectral methods on D1. Even if all sensors were carefully positioned in the center of the sampling frame, differences in the oval field of view of the Greenseeker compared to the circular field of view of the Handyspec may have favored the Greenseeker. Compared to DW and Nup, the Greenseeker exceeded the hyperspectral indices even more for CC. Good CC prediction by the Greenseeker was also confirmed by [46].

Among the established sensor methods, the NDVI constituted on average the best established hyperspectral vegetation index for CC estimation, but was increasingly exceeded by the NDRE for DW and Nup with ongoing plant development. Contour map analysis indicated possible improvements by 11% and 10% compared to NDRE for DW and Nup, respectively, from band combinations with a similar NIR band like the NDRE at 792 or 794 nm but a rightwards shifted red edge band (736 nm) compared to 720 nm. Thus, these indices confirm the simple ratio index R780/R740 that was previously tested for the estimation of Nup in wheat from Zadok’s stage 29 on [65,66] as well as the recommendation of comparable band combinations for estimation of Nup [67,68]. Similar wavelengths were also found to be advantageous for DW estimation [69] and the estimation of absorbed radiation, what was ascribed to the relatively high sensitivity in dense canopies [70].

While the REIP and R780/R740 mostly performed comparably as previously shown for later stages [66], the present results indicate that the REIP may be strongly affected by the soil influence on D1 and D2, but caught up with, and outperformed both NDVI and NDRE on D3 and D4 for both traits, respectively.

Water-related indices combining bands in the NIR range were reported to correlate with biomass and were used for estimating the final yield [71,72]. In this study however, the WBI gave poor correlations and completely failed on D1. The expected advantages of this index concerning overcoming saturation compared to NDVI in dense canopies was mostly not relevant, whereas strong scattering in the higher NIR spectrum beyond approximately 900 nm (Figure 2b) negatively influenced the mean and stability of all correlations found in the contour map in this range.

### 4.3. Estimation of Canopy Cover

Canopy cover is important to be estimated for evaluating early vigor. While the correlations of hyperspectral indices were lower for this trait than for DW and Nup, either spectral unmixing or Greenseeker gave the best correlations per day among the established methods. With the exception of D2, the Greenseeker-NDVI even outperformed the hyperspectral NDVI for this trait. Due to the rather weak light source of this active sensor, it may have become slightly saturated for DW and Nup for canopies with 3D structure at D4 as also found for maize [73]. However, CC was also saturated for later sampling dates, thus that saturation both in target and predictor traits benefitted the active sensor for this trait. In turn, particular vegetation indices including red edge bands and NIR/NIR indices may have been over-sensitive, possibly explaining that unlike for DW or Nup, NDVI still exceeded NDRE for estimating CC.

Interestingly, the early plant vigor index (EPVI) developed for detecting CC [37] performed similarly well as the NDVI exclusively for CC in D3, the day most similar in growth stage to the trials used for the index development, thus indicating specific fitting to growth stage and trait. On most days however, vegetation indices including a green band were the best contour map -indices, which may relate to the HSV-based thresholding process for retrieving CC, that was largely driven by the green/brown contrast. Established hyperspectral vegetation indices did not capture this property adequately so relatively higher improvements were obtained from the contour map indices.

Spectral unmixing was included as method that includes information of the whole spectrum which was related to extreme endmembers soil and dense vegetation. Thus, it could hold the advantage of directly returning values of fractional vegetation cover in the same range of CC. It gave convincing results with the exception of D1. The information included in the very flat canopy spectra was very similar to the soil spectrum on that day. With the soil fraction dominating the spectra at a canopy coverage of only 4.5%, distinct soil scattering was observed. Moreover, the soil surface was still rougher and more heterogeneous between plots, which added additional noise and hindered representative manual selection of reference spectra. Even when advanced endmember selection is performed, soil scattering remains a major draw-back in the VIS and NIR range [30]. Supposing optimal endmember selection, spectral unmixing could catch up with NDVI the following days. Non-linear models might further improve the results [29].

## 5. Conclusions

The results substantiate the hypothesis that RGB imaging can be considered as an alternative to spectral sensing in very early growth stages to determine crop traits. While spectral measurements deliver better results for the later stages, especially at the beginning of vegetation growth they are negatively influenced by high soil reflectance. Main advantages of the RGB methodology are the reasonably low investment and maintenance costs compared to spectral crop sensors that can easily cost some US-$1000 [42]. Furthermore, this technique is easy to use even for unexperienced users and can serve for multiple applications. However, better cameras may be required to achieve sufficient ground resolution depending on the required accuracy when UAVs are used. Even if imaging lost in comparable performance already from Zadoks’s stage 21, hyperspectral sensing will not be the first alternative in many applications whereas the simple and also relatively inexpensive Greenseeker sensor never performed better for DW and Nup until Zadok’s stage 32. However, uncertainties such as less favorable lightning conditions and vibrations from mobile sensor platforms should be addressed. More robust segmentation methods should also be considered. On the other hand, improved precision can be achieved using optimal vegetation indices such as the NDRE. With respect to vegetation indices, the results suggest the use of a normalized difference-index combining 736 with 794 nm for DW and Nup and green VIS bands for estimating CC.

## Figures and Tables

**Table 1:** Sampling dates and growth stages.

Day	Date	Growth Stage
1	03/15/2017	12–13
2	03/28/2017	21
3	04/13/2017	24
4	05/03/2017	32

**Table 2:** List of tested hyperspectral vegetation indices, where *b* refers to the reflectance (*Refl*) in the respective spectral bands.

Vegetation Index	Equation	Reference
ATSAVI	$\frac{1.22*\left(Refl\left[b800\right]-1.22*Refl\left[b670\right]-0.03\right)}{1.22*Refl\left[b800]+Refl[b670\right]+0.23}$	[57]
EPVI	$\frac{Refl\left[b750\right]-Refl\left[b670\right]}{Refl\left[b862\right]}$	[37]
MCARI	$\left(\left(Refl\left[b700\right]-Refl\left[b670\right]\right)-0.2*\left(Refl\left[b700\right]-Refl\left[b550\right]\right)\right)*\frac{Refl\left[b700\right]}{Refl\left[b670\right]}$	[58]
MTCI	$\frac{Refl\left[b750\right]-Refl\left[b710\right]}{Refl\left[b710]-Refl[b680\right]}$	[59]
NDRE	$\frac{Refl\left[b790\right]-Refl\left[b720\right]}{Refl\left[b790]+Refl[b720\right]}$	[16]
NDVI	$\frac{Refl\left[b864\right]-Refl\left[b671\right]}{Refl\left[b864\right]+Refl\left[b671\right]}$	[60]
PRI	$\frac{Refl\left[b531\right]-Refl\left[b570\right]}{Refl\left[b531]+Refl[b570\right]}$	[19]
REIP	$700+40*\frac{Refl\left[b668\right]+\frac{Refl\left[b778\right]}{2}-Refl\left[b698\right]}{Refl\left[b738]-Refl[b698\right]}$	[15]
WBI	$\frac{Refl\left[b900\right]}{Refl\left[b970\right]}$	[17]

**Table 3:** Squared Pearson correlation coefficients (R²) and significance levels (<0.05) for linear relationships of the sensor methods with canopy traits for four measurement dates as well as pairwise relationships of canopy traits. The R² of the best established sensor method per canopy trait is highlighted in bold for each date. Non-significant relationships are not shown. Contour map results refer to the maximum R² extracted from all possible pairwise normalized difference indices. For each trait*sensor method combination, the mean and coefficient of variation (CV) of the R² was calculated across the four days including non-significant relationships, except for NC because the correlations were often not significant. For contour maps, however, the values refer to the maximum of the mean contour map matrix as averaged over four days and the CV of the respective index for accounting for changes in optimum band combinations over time. The ratio of the best contour map correlation to the best correlation found form all other established sensor methods and from the established hyperspectral vegetation indices is given as measure of gain through using optimized vegetation indices. Wavelengths included in contour map indices are indicated. Horizontal table lines group traits from top to bottom into target traits and different types of predictor traits.

		Dry Weight						N Content					
		Date 1	Date 2	Date 3	Date 4	Mean	CV	Date 1	Date 2	Date 3	Date 4	Mean	CV
**N content**				0.31 ***	0.43 ***								
**Dry weight**										0.31 ***	0.43 ***		
**N uptake**		0.99 ***	0.99 ***	0.93 ***	0.91 ***						0.17 *		
**Canopy cover**		0.76 ***	0.65 ***	0.62 ***	0.50 ***	**0.63**	0.17				0.19 **		
**Greenseeker**		0.45 ***	0.53 ***	0.55 ***	0.45 ***	0.50	0.11			0.17 *			
**Spectral unmixing**			0.74 ***	0.70 ***	0.46 ***	0.49	0.65			0.24 **	0.21 **		
**ATSAVI**		0.30 ***	0.71 ***	0.68 ***	0.43 ***	0.53	0.37			0.20 **	0.34 ***		
**EPVI**		0.29 **	0.68 ***	0.63 ***		0.42	0.70			0.17 *	0.2 **		
**MCARI**		0.23 **	0.5 ***	0.27 ***		0.26	0.73						
**MTCI**		0.23 **	0.63 ***	0.76 ***	0.62 ***	0.56	0.41			0.35 ***	0.31 ***		
**NDRE**		0.31 ***	0.75 ***	0.75 ***	0.61 ***	0.61	0.34			0.28 **	0.35 ***		
**NDVI**		0.30 ***	0.72 ***	0.68 ***	0.43 ***	0.53	0.38			0.20 **	0.34 ***		
**PRI**				0.58 ***	0.38 **	0.24	1.18			0.17 *	0.25 **		
**REIP**		0.25 **	0.46 ***	0.77 ***	0.67 ***	0.54	0.43			0.34 ***	0.30 ***		
**WBI**S			0.55 ***	0.71 ***	0.61 ***	0.49	0.56			0.24 **	0.36 ***		
**Contour map**	**R²**	0.42 ***	0.79 ***	0.81 ***	0.74 ***	0.67	0.26	0.37 ***	0.3 ***	0.43 ***	0.49 ***	0.22	0.70
	**Wavelengths**	676	774	826	1030	794		1018	1110	1130	1030	996	
		664	748	746	740	736		978	1108	546	986	924	
**Contour map/best established sensor method**		0.55	1.07	1.05	1.10	1.06				1.23	1.36	0.67	
**Contour map/established hyperspectral vegetation index**		1.35	1.05	1.05	1.10	1.11				1.23	1.36	0.67	
		**N uptake**						**Canopy cover**					
		**Date 1**	**Date 2**	**Date 3**	**Date 4**	**mean**	**CV**	**Date 1**	**Date 2**	**Date 3**	**Date 4**	**mean**	**CV**
**N content**					0.17 *						0.19 **		
**Dry weight**		0.99 ***	0.99 ***	0.93 ***	0.91 ***			0.76 ***	0.65 ***	0.62 ***	0.5 ***		
**N uptake**								0.78 ***	0.67 ***	0.69 ***	0.48 ***		
**Canopy cover**s		0.78 ***s	0.67 ***	0.69 ***	0.48 ***	0.66	0.19						
**Greenseeker**		0.45 ***	0.54 ***	0.53 ***	0.46 ***	0.50	0.09	0.49 ***	0.42 ***	0.73 ***	0.70 ***	0.59	0.26
**Spectral unmixing**			0.75 ***	0.66 ***	0.40 ***	0.47	0.65		0.56 ***	0.63 ***	0.70 ***	0.49	0.59
**ATSAVI**		0.34 ***	0.72 ***	0.66 ***	0.31 ***	0.51	0.42	0.25 **	0.52 ***	0.68 ***	0.56 ***	0.50	0.36
**EPVI**		0.32 ***	0.69 ***	0.62 ***		0.41	0.74	0.23 **	0.48 ***	0.68 ***	0.35 ***	0.44	0.44
**MCARI**		0.27 **	0.51 ***	0.31 ***		0.30	0.69	0.21 **	0.36 ***	0.65 ***	0.35 ***	0.39	0.47
**MTCI**		0.25 **	0.64 ***	0.66 ***	0.52 ***	0.52	0.36	0.14 *	0.45 ***	0.47 ***	0.40 ***	0.37	0.42
**NDRE**		0.34 ***	0.76 ***	0.69 ***	0.50 ***	**0.57**	0.33	0.25 **	0.53 ***	0.62 ***	0.50 ***	0.48	0.33
**NDVI**		0.34 ***	0.73 ***	0.66 ***	0.32 ***	0.51	0.42	0.25 **	0.53 ***	0.68 ***	0.56 ***	0.51	0.36
**PRI**				0.57 ***	0.30 ***	0.22	1.23			0.71 ***	0.56 ***	0.34	1.03
**REIP**		0.27 **	0.48 ***	0.68 ***	0.58 ***	0.50	0.35	0.16 *	0.37 ***	0.48 ***	0.43 ***	**0.36**	**0.39**
**WBI**			0.56 ***	0.66 ***	0.51 ***	0.46	0.54		0.45 ***	0.62 ***	0.39 ***	0.38	0.60
**Contour map**	**R²**	0.45 ***	0.81 ***	0.75 ***	0.68 ***	0.63	0.23	0.42 ***	0.66 ***	0.74 ***	0.74 ***	0.55	0.22
	**Wavelengths**	792	1068	758	1018	792		584	1134	556	1108	584	
		736	544	754	994	736		582	580	552	726	582	
**Contour map/best established sensor method**		0.58	1.08	1.09	1.17	0.96		0.86	1.18	1.01	1.06	0.94	
**Contour map/established hyperspectral vegetation index**		1.32	1.07	1.09	1.17	1.10		1.68	1.25	1.04	1.32	1.09	
